# Booster vaccination against tetanus and diphtheria: insufficient protection against diphtheria in young and elderly adults

**DOI:** 10.1186/s12979-016-0081-0

**Published:** 2016-09-05

**Authors:** Marco Grasse, Andreas Meryk, Michael Schirmer, Beatrix Grubeck-Loebenstein, Birgit Weinberger

**Affiliations:** 1 Institute for Biomedical Aging Research, Universität Innsbruck, Rennweg 10, A-6020 Innsbruck, Austria; 2 Department of Internal Medicine VI, Medical University Innsbruck, Innsbruck, Austria

**Keywords:** Booster vaccination, Tetanus, Diphtheria, Adults, Elderly

## Abstract

We have recently demonstrated that single shot vaccinations against tetanus and diphtheria do not lead to long-lasting immunity against diphtheria in elderly persons despite administration at 5 year intervals. In the present study we have immunized a group of young adults against tetanus and diphtheria to compare the pre- and 28 days post-vaccination immune responses in the young group with results of the same vaccination performed in an elderly group of a previous study. We also studied protection in both groups 5 years after vaccination. We compared antibody titers at all three time points and also analyzed the T cell responses in both age groups 5 years after vaccination.

Before vaccination 9 % of the elderly persons were not protected against tetanus, and 48 % did not have protection against diphtheria. In the young group all participants were protected against tetanus, but 52 % were also unprotected against diphtheria before vaccination. 28 days after vaccination 100 % of all participants had protective antibody concentrations against tetanus and only a small percentage in each age group (<10 %) was unprotected against diphtheria. 5 years later, 100 % of both cohorts were still protected against tetanus, but 24 % of the young and 54 % of the elderly group were unprotected against diphtheria. Antibody concentrations against diphtheria measured by ELISA correlated well with their neutralizing capacity. T cell responses to tetanus and diphtheria did not differ between young and old persons. We conclude that booster vaccinations against tetanus and diphtheria according to present recommendations provide long-lasting protection only against tetanus, but not against diphtheria, independently of age. In elderly persons, the level of protection is even lower, probably due to intrinsic age-related changes within the immune system and/or insufficient vaccination earlier in life.

**Electronic supplementary material:**

The online version of this article (doi:10.1186/s12979-016-0081-0) contains supplementary material, which is available to authorized users.

## Introduction

Immunization is one of the most successful and cost-effective health interventions against infectious diseases. Since its introduction the mortality and incidence rate of many diseases has been reduced dramatically, as for instance for measles, rubella or diphtheria [1, 2]. Tetanus and diphtheria vaccines, which contain the bacterial toxoids in combination with aluminum salts, are among the most frequently used vaccines worldwide. The current WHO’s vaccination recommendation against tetanus and diphtheria comprises a primary vaccination series during childhood and regular booster vaccinations throughout life [3, 4]. In Austria, vaccination against tetanus and diphtheria is recommended every 10 years for adults and every 5 years for people above 60 years of age [5]. However, we and others have repeatedly reported insufficient antibody (Ab) concentrations against both antigens (specific Abs <0.1 IU/ml) in adults, particularly in the elderly [6–12]. The proportion of unprotected individuals varied between 2 and 60 % for tetanus and between 29 and 70 % for diphtheria. Lack of protection in elderly persons is suggested to be due to intrinsic changes within the immune system [13], but may also be due to lack of, or incomplete primary immunization or the absence of regular booster shots.

We immunized an elderly cohort in 2005 providing the participants with one shot of Repevax® and measured pre- and post-vaccination Ab concentrations [11]. Although protection against tetanus was sufficient in the majority of participants before and after vaccination, protection levels against diphtheria were very low before but almost 100 % 28 days after vaccination. According to Austrian recommendations, the participants were invited for a booster shot (Boostrix®) 5 years later, in 2010. Surprisingly, protection against diphtheria was once again low, almost as low as at the time of the first recruitment in 2005, demonstrating that our initial vaccination strategy had failed to induce long-lasting immunity against diphtheria in this age group [12]. In order to find out whether the short-lasting immunity observed in old people was only due to their advanced age, we recruited a young cohort and administered one dose of BoostrixPolio® to compare pre- and post-immunization Abs with the elderly cohort. After another 5 years, in 2015, Abs and T cell responses against tetanus and diphtheria were analyzed in both age groups. We demonstrated that protection against tetanus was 100 % in both age groups, but that protection against diphtheria was lost in 54 % of the elderly group. Interestingly, 24 % of the young cohort did also not have protective Ab concentrations against diphtheria, in spite of an only 5 year interval since the last vaccination.

## Material and methods

### Study cohorts and protocol

87 healthy, elderly adult volunteers (median age 71 years, range 66–92 years; 47 females), who had received one dose of Repevax® (Sanofi Pasteur MSD) in 2005 [11], were vaccinated once more against tetanus, diphtheria and pertussis (Boostrix®, GlaxoSmithKline) 5 years later in 2010 [12]. Pre- and 28 days post-vaccination Ab concentrations of the 2010 vaccination were now compared with corresponding data from a group of 46 healthy, young adult volunteers (median age 29 years, range 24–40 years; 29 females) which was recruited in 2010 and received one booster vaccination against tetanus, diphtheria, pertussis and polio (Boostrix®-Polio, GlaxoSmithKline). Their previous booster shot was well documented and dated back more than 10 years. All vaccinations were performed in accordance with the official recommendations by the Austrian health authorities [5]. Young and old participants were recruited from the general population and all participants of the old cohort were community-dwelling. Persons with chronic viral infection (Human Immunodeficiency virus, Hepatitis B virus, Hepatitis C virus), transplant recipients and patients under immunosuppressive or chemotherapy were not included in the study. Routine laboratory parameters (liver and kidney function, blood count) were determined at the time point 2010 and all participants were shown to be in good health [12]. All participants were invited back 5 years later, in 2015, for the analysis of the level of protection against tetanus and diphtheria by measuring Abs as well as T cell function. Due to various reasons (such as unwillingness to participate, change of address, ill health, death or meeting one of the exclusion criteria), only 27 elderly and 17 young adults could be studied in 2015. Exclusion criteria were the same for both groups in 2010 and 2015, namely chronic viral infections (HIV, HBV, HCV), transplant reception, cancer as well as immunosuppressive therapy. Pregnant or lactating females were also excluded from the study. We checked whether the participants re-recruited in 2015 were representative for the original larger cohorts, and found that neither age nor sex were different. We also compared pre- and post-vaccination Ab concentrations measured in 2010 between the total cohort and the sub-cohorts available for further analysis in 2015 and did not find any statistically significant difference.

### Preparation of plasma and peripheral blood mononuclear cells (PBMCs)

Heparinized blood was drawn from the arm vein and fractionized by density gradient centrifugation using Ficoll-Paque™ Plus (GE Healthcare) to collect plasma and PBMCs. PBMCs were used freshly and plasma was stored at −20 °C.

### Determination of Ab concentrations by ELISA

Microtiter plates were coated with 1 μg/ml tetanus or diphtheria toxoid (Statens Serum Institute) and blocked with 0.01 M Glycin. Plasma samples were tested in duplicates. Peroxidase-labeled rabbit anti-human IgG Ab (Chemicon/Millipore) was used as secondary Ab. IgG Abs were quantified in IU/ml using standard human anti-tetanus and anti-diphtheria sera (National Institute for Biological Standards and Control). The detection limit of the assays used was 0.01 IU/ml, and values below this concentration were set to 0.005 IU/ml for calculation of geometric mean concentrations (GMC). Ab concentrations above 0.1 IU/ml were considered as protective [3, 4].

### Determination of Abs against diphtheria by neutralization assay

Plasma samples were incubated for one hour with 0.1 M 2-mercaptoethanol in order to eliminate IgM Abs. After heat inactivation at 56 °C for 30 min serial dilutions ranging from 1:20 to 1:4800 were prepared using Dulbecco’s modified eagle’s medium (Sigma Aldrich) containing 2 % fetal calf serum (FCS; Sigma Aldrich) and 1 % Penicillin-Streptomycin (Sigma Aldrich). The pre-diluted samples were incubated with diphtheria toxin (8 ng/ml; Sigma Aldrich) for 90 min. This mixture was transferred into 96-well tissue culture plates seeded with 15.000 Vero cells 24 h earlier. After 48 h Vero cells were stained with crystal violet. Living cells were stained violet, indicating that the Abs were able to neutralize the diphtheria toxin. All incubations were performed at 37 °C and 5 % CO_2_.

### Flow cytometric analysis of cytokine production

Cytokine production of antigen-specific CD4^+^ T cells was induced by stimulation of PBMCs with 10 μg/ml tetanus- or diphtheria-toxoid at 37 °C for 6 h with 10 μg/ml Brefeldin A added after the first hour of stimulation. PBMCs were washed with PBS and stained with Zombie Violet™ Fixable Viability dye (Biolegend) for 20 min at RT and washed with FACS buffer (PBS + 2 mM EDTA + 2 % FCS + NaN_3_). Cells were stained with anti-CD3-BV510 (clone: UCHT1; BD Biosciences), anti-CD4-PE-Cy™7 (clone: SK3; BD Biosciences) and anti-CD45RO-PerCP-Cy™5.5 (clone: UCHL1; BD Biosciences) for 20 min at 4 °C. After washing with FACS buffer, cells were fixed and permeabilized using the BD Cytofix/Cytoperm™ kit (BD Biosciences) following manufacturer’s instructions. Cells were then stained with anti-IFN-γ-FITC (clone: B27; BD Biosciences), anti-TNF-α-PE (Mab11; BD Biosciences), anti-IL2-APC (clone: MQ1-17H12; BD Biosciences), anti-IL4-PE (clone: 8D4-8; BD Biosciences), anti-IL10-APC (clone: JES3-19 F1; Biolegend), anti-IL17-PE (clone: eBio64DEC17; eBioscience), anti-IL21-Alexa Fluor®647 (clone: 3A3-N2.1; BD Biosciences), anti-TGF-β1-PE (clone: TW4-9E7; BD Biosciences) and anti-GM-CSF- Alexa Fluor®647 (clone: BVD2-21C11; BD Biosciences) for 30 min in BD Cytoperm™ (BD Biosciences) at 4 °C. After washing with PBS, cells were measured using the FACS canto II cytometer (BD) and analyzed using FlowJo software (V 10.0.7.). CD4^+^ memory cells were gated as CD3^+^CD4^+^CD45RO^+^. The unstimulated controls were subtracted from the antigen-specific samples for each cytokine and donor.

### Statistical analysis

Group wise comparisons for tetanus- and diphtheria-specific Abs as well as for antigen-specific cytokine-producing T cells were performed using the Wilcoxon test. The Wilcoxon-signed rank test was applied for paired data and the Wilcoxon rank-sum test for unpaired data.

The frequency of persons protected/unprotected against tetanus and diphtheria in the young and the elderly group was compared using the chi-square test.

Spearman rank correlations were applied to study relations between neutralizing capacity and total concentrations of diphtheria-specific Abs, Ab concentrations and the time since the last vaccination, as well as to study the relationship between Abs and cytokine production in T cells.

The level of significance for all tests was α = 0.05. Accordingly, the critical value for the chi-square test was *x*^2^ = 3.84.

SPSS version 23 (SPSS Inc., Chicago, USA) was used for all statistical analyzes.

## Results

### Antibody concentrations against tetanus and diphtheria in young and elderly persons measured by ELISA

**Fig 1:**
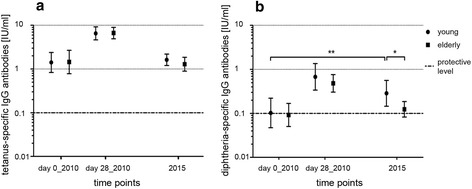
Tetanus- and diphtheria-specific Ab concentrations measured by ELISA. Tetanus- **a** and diphtheria- **b** specific Ab concentrations of young and elderly adults are shown as geometric mean concentrations (GMCs) ± 95 % confidence interval. Ab concentrations of young and old donors were compared before (day 0_2010), 4 weeks after (day 28_2010) as well as 5 years after (2015) a tetanus and diphtheria booster shot. Wilcoxon-signed rank test was applied for comparisons within the age groups (paired data) and the Wilcoxon rank-sum test was applied for comparisons among the age groups (unpaired data). * *p* = 0.0266 (young vs. old in 2015) ** *p* = 0.0052 (young pre-vaccination 2010 vs. 2015)

The geometric mean concentrations (GMCs) of tetanus- and diphtheria-specific Abs at three different time points are shown in Fig. 1: Pre-vaccination Ab concentrations in 2010, post-vaccination Ab concentrations on day 28 in 2010 and 5 years later in 2015. For tetanus (Fig. 1a) Ab concentrations were generally high, well above the protective level, and were not different in the two age groups. For diphtheria (Fig. 1b), GMCs were around the protective level in both age groups before vaccination in 2010, but well in the protective range 28 days after vaccination. Five years later, expectedly Ab concentrations had decreased in both age groups, but were significantly higher in the young than in the elderly cohort. In 2015 Ab concentrations in the young group were also higher than the pre-vaccination values in 2010. In contrast, Ab concentrations in the old cohort were back to the pre-vaccination level in 2010 (Fig. 1b).

### Percentage of young and elderly persons protected against tetanus and diphtheria

**Fig 2:**
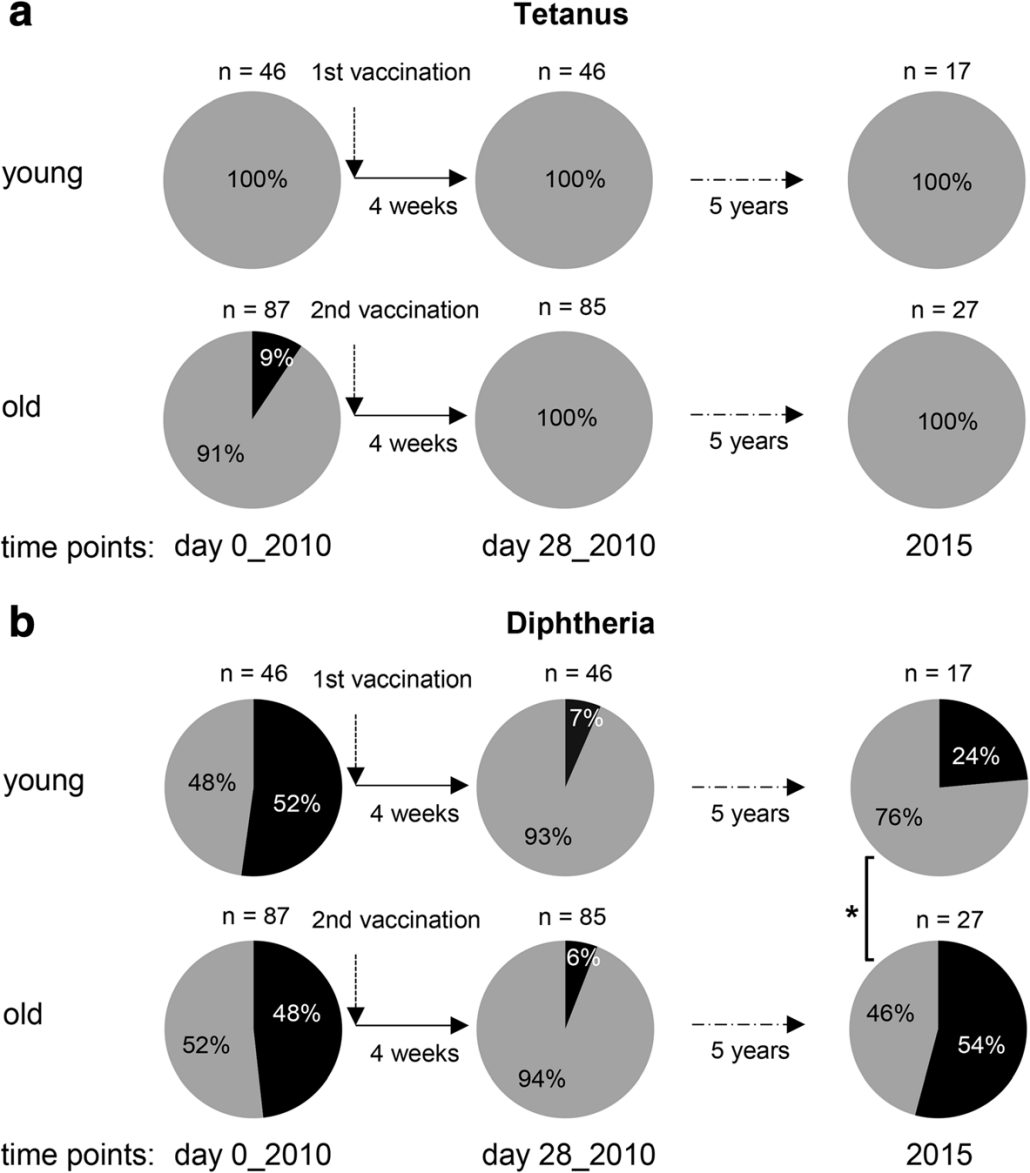
Level of protection against tetanus and diphtheria. Tetanus- **a** and diphtheria- **b** specific Ab concentrations from young and elderly adults were measured by ELISA. A concentration above 0.1 IU/ml was considered as protective. Frequencies of protected (grey) and unprotected (black) individuals are shown. Plasma samples were collected before (day 0_2010), 4 weeks after (day 28_2010) as well as 5 years after (2015) the booster shot. Chi-squared test was applied for statistical analysis. * *x* ^2^ = 4.36 (x^2^_crit_ = 3.84)

Corresponding to the high Ab concentrations against tetanus, 100 % of both age groups were protected against this antigen. A small proportion of elderly persons who had been unprotected before vaccination in 2010 were protected after the vaccination and were still protected 5 years later (Fig. 2a).

The situation was different for diphtheria (Fig. 2b). In both age groups, about half of the cohort did not have protective Ab concentrations before vaccination in 2010. Most participants from both age groups had protective Ab concentrations 4 weeks after vaccination, but a small number of persons (<10 %) from each age group remained unprotected. After 5 years, the percentage of unprotected persons had dropped in both age groups, with 54 % of the elderly group and 24 % of the young group being unprotected, which was a significant difference (Fig. 2b).

### Functional analysis of diphtheria-specific Abs

**Fig 3:**
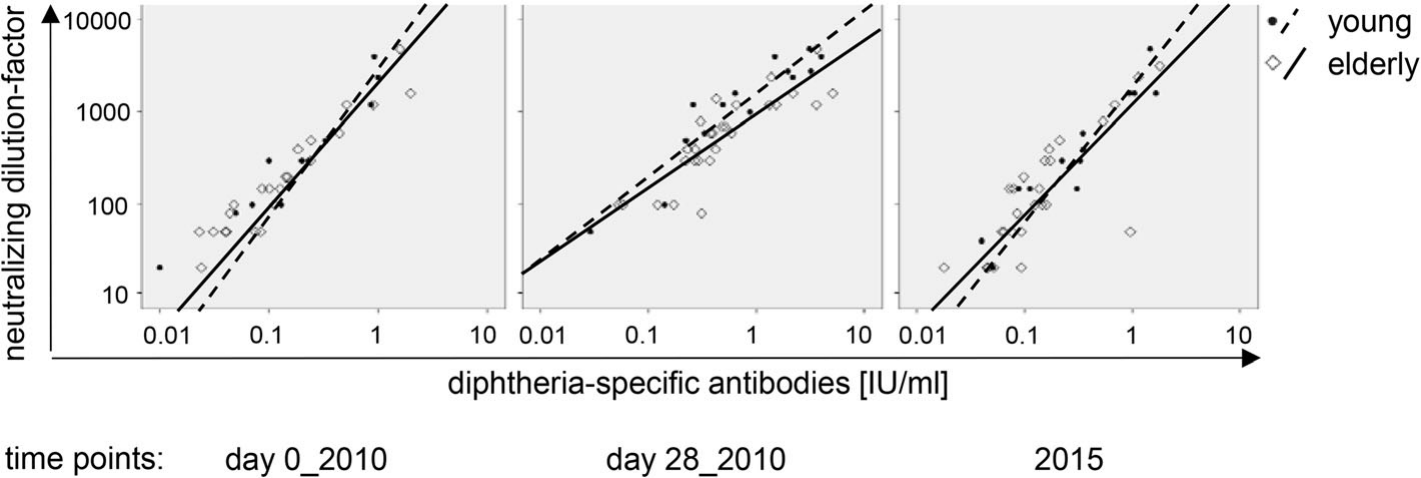
Diphtheria-specific Abs measured by ELISA and neutralizing assay. The concentrations of diphtheria-specific Abs measured by ELISA are shown in correlation to the highest plasma dilution factor able to neutralize diphtheria toxin (8 ng/ml). Diphtheria-specific Abs were compared in samples taken before (day 0_2010), 4 weeks after (day 28_2010) as well as 5 years after (2015) the booster shot. Elderly adults (*n* = 27) are represented by empty diamonds and solid trend-lines, young adults (*n* = 17) by filled dots and dashed trend-lines. Spearman rank correlation was applied and correlation coefficients and *p*-values were determined

In view of the fact that Ab concentrations against diphtheria were quite low in some persons, we analyzed the functionality of diphtheria-specific Abs in young and older adults. We therefore measured the neutralizing capacity of diphtheria-specific Abs from 27 elderly and 17 young adults at three time points: immediately before and 28 days after vaccination (2010) and 5 years later (2015). The neutralizing capacity and diphtheria-specific Ab concentrations measured by ELISA were highly correlated (*p* <0.0001, r_s_ >0.821 in both age groups at all time points, Fig. 3).

### The impact of time since the last vaccination on Ab concentrations

**Fig 4:**
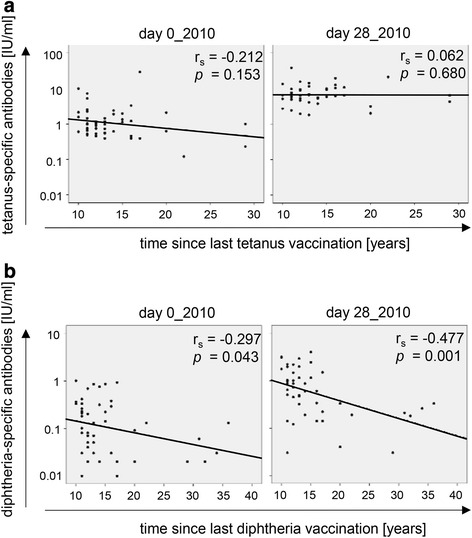
Impact of vaccination history on Ab concentrations in young persons. Tetanus- **a** and diphtheria- **b** specific Ab concentrations are shown in correlation to the time since the last vaccination before recruitment for this study. Correlations were calculated using Ab concentrations assessed in young adults (*n* = 46) before (day 0_2010) and 4 weeks after (day 28_2010) the tetanus/diphtheria vaccination in 2010. Spearman rank correlation coefficients (r_s_) and *p*-values are indicated

Recent vaccination history was synchronized for the older cohort, as they had received tetanus and diphtheria vaccinations in the context of our studies in 2005 and 2010 [11, 12]. In contrast, the time since the last vaccination before the recruitment in 2010 varied considerably within the young group. Correlations between pre- and post-vaccination Ab concentrations in 2010 and the time since the last vaccination were therefore only analyzed in the young group (Fig. 4). For tetanus there was no correlation between Ab concentrations and the time point of the last vaccination (Fig. 4a). In contrast, for diphtheria, there was a significant correlation between Ab concentrations and the time since the last vaccination (Fig. 4b). This correlation was most pronounced for the Ab concentrations 28 days after vaccination, indicating that regular booster vaccinations against diphtheria are important not only for the maintenance of Ab levels, but also for the success of booster vaccinations.

### T cell responses to tetanus and diphtheria

**Fig 5:**
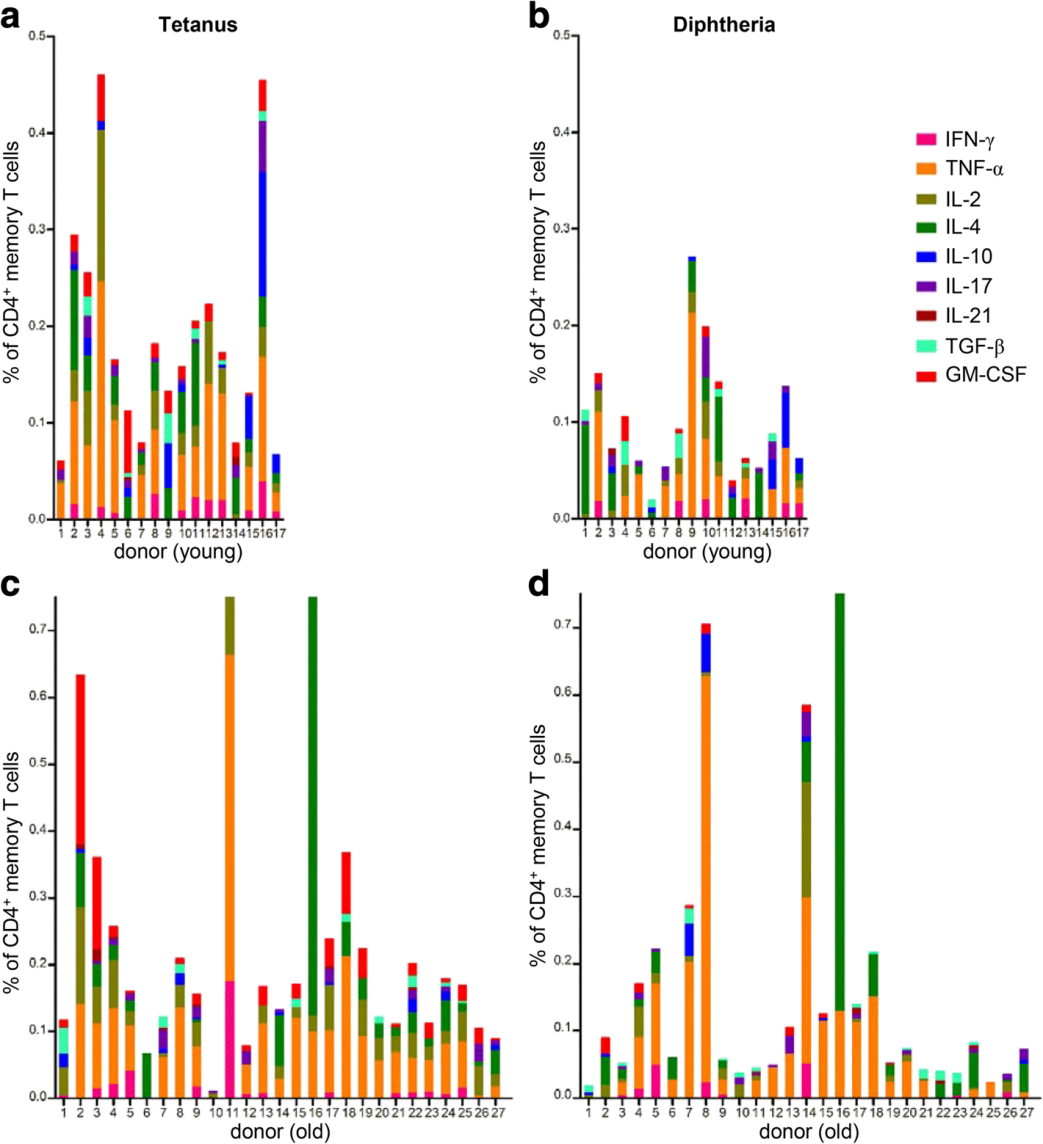
Cytokine production by tetanus- and diphtheria-specific CD4^+^ memory T cells. PBMCs were stimulated for 6 h with 10 μg/ml tetanus (**a c**) or 10 μg/ml diphtheria toxoid (**b d**) in the presence of Brefeldin A (5 h). Frequencies of CD4^+^ memory T cells producing IFN-γ, TNF-α, IL-2, IL-4, IL-10, IL-17, IL-21, TGF-β and GM-CSF were measured by flow cytometry. Data from young (**a b**; *n* = 17) and elderly (**c d**; *n* = 27) adults are shown. A representative example for the gating strategy is shown in Additional file 2: Figure S1

In a previously published study on elderly adults [12] we found a weak correlation between IL-5-producing T cells measured by Elispot and diphtheria-specific Abs. In the present study, a detailed analysis of cytokine production by CD4^+^ memory cells was performed at the 2015 time point using flow cytometry. The production of 9 cytokines (IFN-γ, TNF-α, IL-2, IL-4, IL-10, IL-17, IL-21, TGF-β, GM-CSF) following in vitro stimulation of PBMCs with tetanus (Fig. 5a and c) and diphtheria (Fig. 5b and d) toxoid was analyzed, and was found to be similar in young (Fig. 5a and b) and elderly (Fig. 5c and d) adults. The production of more than one cytokine was detected in CD4^+^ memory cells of all donors. Tetanus-specific T cells of young and old donors produced 5.8 ± 1.2 (mean ± SD) and 5.4 ± 1.8 cytokines, respectively (n.s.; Wilcoxon rank-sum test). 4.2 ± 1.0 and 4.0 ± 1.4 cytokines were detected after stimulation with diphtheria toxoid in T cells of young and old donors, respectively (n.s.; Wilcoxon rank-sum test). The frequency of all antigen-specific cytokine-producing T cells was similar in both age groups for tetanus and diphtheria (n.s.; Wilcoxon rank-sum test). Correlations between Ab concentrations and cytokine production were also performed (Table 1). There was no correlation between tetanus Ab concentrations and cytokine production by tetanus-specific CD4^+^ memory T cells, whereas a weak correlation was found between diphtheria-specific Abs and diphtheria-specific IL-2-, IL-21- and GM-CSF-producing CD4^+^ memory T cells. All these correlations were performed with pooled data from both age groups (*n* = 44) due to the low sample sizes in each group. Results were similar when both age groups were analyzed separately, but did not reach statistical significance.

**Table 1:** Correlations between antigen-specific cytokine-producing CD4^+^ memory T cells and Abs

	Tetanus		Diphtheria	
Cytokine	r_s_	*p*-value	r_s_	*p*-value
IFN-γ	−0.059	0.715	0.195	0.204
TNF-α	−0.078	0.626	0.214	0.180
IL-2	0.067	0.678	0.398	0.010
IL-4	−0.090	0.563	0.067	0.665
IL-10	0.001	0.996	0.091	0.570
IL-17	−0.223	0.160	−0.110	0.494
IL-21	−0.018	0.913	−0.309	0.049
TGF-β	0.054	0.739	0.200	0.192
GM-CSF	−0.019	0.906	0.347	0.026

Correlations were performed with pooled data from both age groups (*n* = 44)

*Abbreviation*: *r*_*S*_ spearman’s rank correlation coefficient

## Discussion

In a previous study, we investigated the level of protection against tetanus and diphtheria in an elderly population and analyzed the immune response to tetanus and diphtheria following two doses of vaccine applied at a 5 year interval [12]. The level of protection against tetanus was much higher than the one against diphtheria at both time points and reached almost 100 % protection 4 weeks after the booster shots. With the applied vaccination strategy we followed official Austrian recommendations according to which persons of more than 60 years of age should receive a booster vaccination every 5 years. It was surprising that even after this relatively short period of time almost half of the cohort had lost protective Ab concentrations against diphtheria and were again unprotected 5 years after the first vaccination. Protection could be re-obtained in 94 % of the cohort 28 days after a second shot of diphtheria vaccine. It was the goal of the present study to re-analyze the cohort after another 5 years and additionally to compare them with a young cohort in order to clarify the role of age-related intrinsic changes within the immune system.

We now demonstrate that in spite of having applied the tetanus/diphtheria vaccine twice, diphtheria-specific Ab concentrations had again dropped to unprotective levels in more than half of the elderly cohort. This was not the case for tetanus, against which 100 % of the elderly cohort were now protected. Surprisingly, the situation was similar, although to a lesser extent, in the young group, in which 24 % were unprotected despite the fact that the last booster shot had been applied only 5 years earlier. Diphtheria vaccination is recommended every 10 years for young adults. It can be speculated that Ab concentrations will drop below protective levels in an even larger proportion of the young cohort until they receive the next booster vaccination. Similar to the elderly cohort, 100 % of the young persons had protective Ab concentrations against tetanus.

These results suggest that, although age-related changes in the immune system may play some role, diphtheria vaccination does not provide satisfactory results at any age. This may be due to several reasons: As depicted in Fig. 1, in both age groups the levels of diphtheria-specific Abs were one order of magnitude lower than of tetanus-specific Abs. The same finding has been reported by other groups [14–16]. Low Ab concentrations against diphtheria following booster vaccination compared to tetanus are presumably due to the fact that vaccines used for booster vaccination in adults contain much less diphtheria toxoid than the vaccines used for primary immunization. The reduction of the amount of diphtheria toxoid per dose was originally implemented because of reported side effects after vaccination with higher diphtheria concentrations [17–19]. However, these reports date back quite a while and it is presently not clear whether improved production and purification processes would make a higher diphtheria toxoid dose possible without adverse events. It might thus be a possibility to evaluate higher diphtheria toxoid doses in adults in order to achieve longer-lasting immunity. As the neutralizing activity of diphtheria Abs is not impaired, neither in young nor in elderly persons, it would be sufficient to increase the titers to achieve longer-lasting immunity. Similar Ab quality in young and old persons has also been described for TBE and influenza vaccination [20, 21].

In this context it was also of interest that in the young group the response to the diphtheria booster shot was improved when the last booster immunization did not date back too long. This finding demonstrates the importance of regular booster immunizations throughout adulthood. However, awareness of this necessity seems to be insufficient among medical staff and young adults, considering the fact that the last vaccination against diphtheria dated back up to 36 years in our young cohort. This means that some participants had never received an adult booster shot.

Documentation of the primary vaccination is frequently poor for adults and particularly for the elderly as shown in several studies [7, 9]. It is therefore not clear, whether all participants had received a complete primary immunization. At first enrollment the date of the last vaccination against diphtheria was documented for only 47 % of the participants [12]. Whether single booster shots with reduced diphtheria content even at relatively short intervals might eventually lead to long-lasting protection under these circumstances seems doubtful in view of our results, which demonstrate that even two shots at a 5 year interval cannot retain protective immunity for the duration of this time period in elderly persons.

How can this dilemma be solved? Improved vaccines specifically tailored for the needs of the 50+ generation could be designed, but which requirements should such vaccines fulfill? A stronger effect on T cells might be one strategy to improve Ab responses. Diphtheria-specific T cell responses were not compromised in our elderly cohort, and results showed weak but significant correlations between T cell cytokines and Ab concentrations for diphtheria; however, no correlations were seen for tetanus, indicating that T cell help might only be needed in the case of poor Ab responses. Although aluminum salts have been used for almost one century as adjuvants in order to improve the immunogenicity of protein vaccines [22], the detailed mode of action is unclear and still a topic of research [23, 24]. Thus, aluminum adjuvants may not be the ideal candidates to achieve a better T cell help. It was of interest that GM-CSF was among the cytokines, which showed a positive correlation with diphtheria Ab concentrations. GM-CSF is recognized as an adjuvant candidate and has been tested in combination with several types of immunization. In humanized mice, GM-CSF treatment has been shown to significantly improve antigen-specific Ab responses following immunization with H5N1 influenza vaccine [25]. GM-CSF has also been successfully used to overcome immune tolerance in a mouse model for therapeutic vaccination against hepatitis B [26]. Administration of GM-CSF improved immune responses to crude *leishmania* antigen vaccine in healthy adults [27]. GM-CSF is used for dendritic cell (DC) maturation in vitro and may also be used to improve this process in the elderly, as age-related defects in DC function and maturation have been described (reviewed in [28, 29]). This might trigger the T cell/B cell axis in order to achieve better Ab production. Alternatively, GM-CSF-producing memory T cells could also be specifically stimulated by other new adjuvants.

In summary, we would like to point out that tetanus vaccination following present recommendations seems to yield satisfactory results, which is not the case for diphtheria. As a minimum measure, regular booster shots should be applied in young adults, a strategy which is, however, unlikely to be successful in elderly persons. Therefore efforts to generate new vaccines and/or vaccination strategies are needed to elicit better protection in the elderly. This may also be necessary for the pertussis component of the vaccine which has been studied in the past [11], but has not been included in the present study.

## Additional files

Additional file 1: Table S1. Complete raw data. (XLSX 30 kb) Additional file 2: Figure S1. Gating strategy for the analysis of the intracellular stainings. A representative example of CD4^+^ memory T cells producing IFN-γ, TNF-α, IL-2, IL-4, IL-10, IL-17, IL-21, TGF-β and GM-CSF after 6 h of tetanus toxoid (10 μg/ml) stimulation is shown (TIF 865 kb)

## Abbreviations

Ab: Antibody
DC: Dendritic cell
FCS: Fetal calf serum
GMC: Geometric mean concentration
PBMCs: Peripheral blood mononuclear cells
