# Supplementary figures and images for: Booster vaccination against tetanus and diphtheria: insufficient protection against diphtheria in young and elderly adults
Source: Immun Ageing. 2016 Sep 5;13(1):26. doi: 10.1186/s12979-016-0081-0 (PMC5011835; doi:10.1186/s12979-016-0081-0)

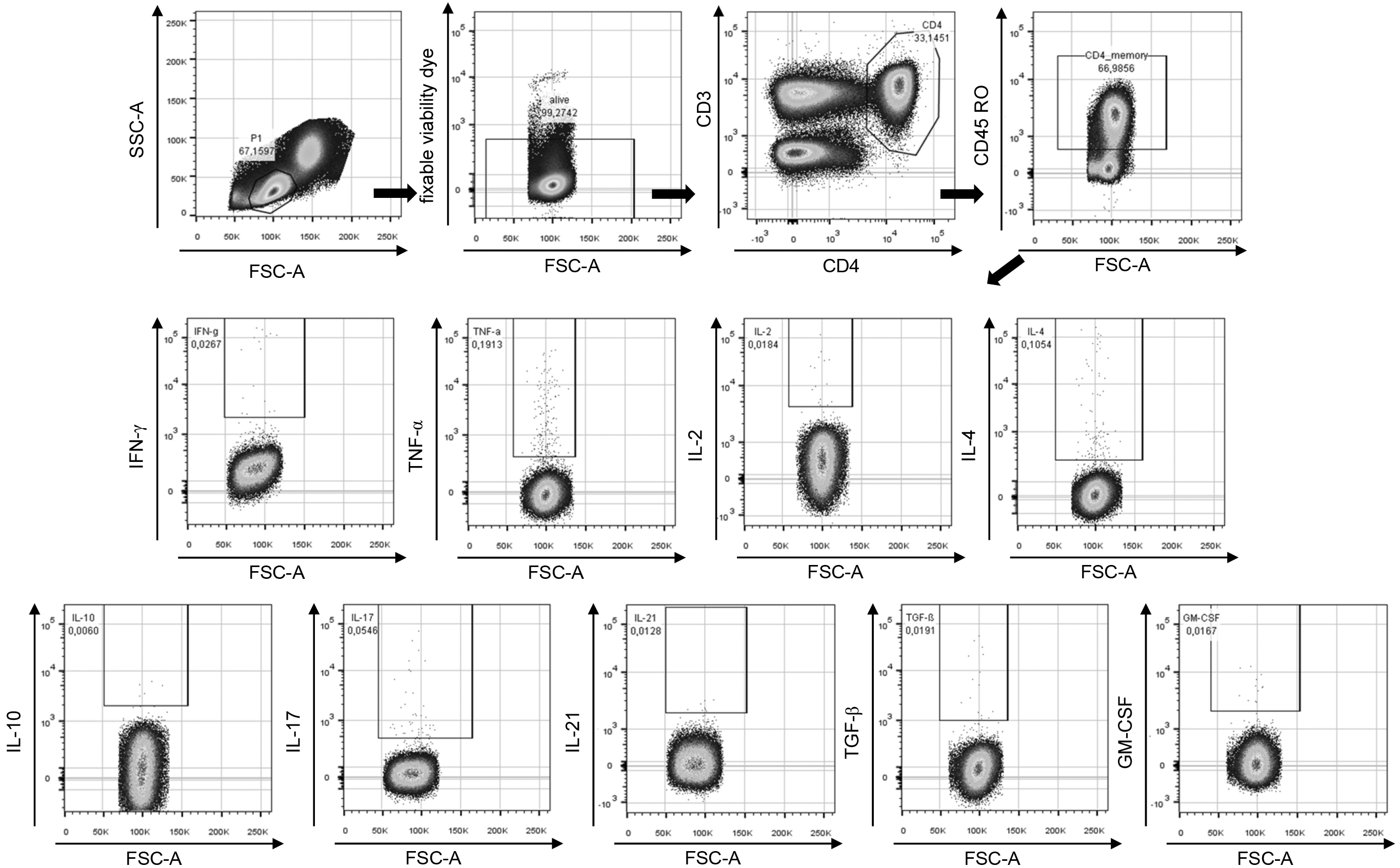

Supplement: Additional file 2: Figure S1. — Gating strategy for the analysis of the intracellular stainings. A representative example of CD4+ memory T cells producing IFN-γ, TNF-α, IL-2, IL-4, IL-10, IL-17, IL-21, TGF-β and GM-CSF after 6 h of tetanus toxoid (10 μg/ml) stimulation is shown (TIF 865 kb) [file 12979_2016_81_MOESM2_ESM.tif]
